# Reading Shakespeare Sonnets: Combining Quantitative Narrative Analysis and Predictive Modeling —an Eye Tracking Study

**DOI:** 10.16910/jemr.12.5.2

**Published:** 2019-03-27

**Authors:** Shuwei Xue, Jana Lüdtke, Teresa Sylvester, Arthur M. Jacobs

**Affiliations:** Freie Universität Berlin, Germany; Dahlem Institute for Neuroimaging of Emotion (D.I.N.E.), Berlin, Germany; Center for Cognitive Neuroscience Berlin (CCNB), Berlin, Germany

**Keywords:** Literary reading, eye movements, eye tracking, QNA, predictive modeling

## Abstract

As a part of a larger interdisciplinary project on Shakespeare sonnets’ reception (1, 2), the present study analyzed the eye movement behavior of participants reading three of the 154 sonnets as a function of seven lexical features extracted via Quantitative Narrative Analysis (QNA). Using a machine learning-based predictive modeling approach five ‘surface’ features (word length, orthographic neighborhood density, word frequency, orthographic dissimilarity and sonority score) were detected as important predictors of total reading time and fixation probability in poetry reading. The fact that one phonological feature, i.e., sonority score, also played a role is in line with current theorizing on poetry reading. Our approach opens new ways for future eye movement research on reading poetic texts and other complex literary materials(3).

## Introduction

When was the last time you read a poem, or a piece of literature? The answer of many people might well be ‘today’ or ‘yesterday’. Even though reading literature may no longer count among the essential activities of people’s leisure time, it still has a significant number of benefits in promoting, for example, general and cross-cultural education, social cognition or cognitive development (4, 5, 6 ,7). However, within the fields of reading and eye tracking research, single words or single sentences from non-literary materials appear to be the most extensively investigated text materials (8 ,9 ,10). Although psycholinguistic features, e.g., *word length* or *word frequency*, work differently in a connected text context (11, 12, 13), empirical research using natural materials like narrative texts or poems are quite rare and the majority of studies on literary works confine to text-based qualitative aspects (e.g., ‘close reading’). Reading research seems to be experiencing difficulty to open itself for empirical studies focusing on more natural and ecologically valid reading acts, as recently admonished by several researchers (14, 15, 13).

With the present study, we aim to explore which and how psycholinguistic features influence literary reading (e.g., some famous poems) by analyzing participants’ eye movement behavior which provides a valid measure of moment-to-moment comprehension processes (16, 17). To achieve our objective, we faced two major challenges: dissecting the complex literary works into measurable and testable features and applying computational methods which can handle the intercorrelated psycholinguistic features and the nonlinear relationship between them and reading behavior. In the following sections, we expound the two challenges separately, and at the end put forward our hypotheses.

### Quantitative Narrative Analysis (QNA)

As we all know, natural texts mostly show a high level of complexity. They are built of single words that can be characterized by more than 50 lexical and sublexical features influencing their processing in single-word recognition tasks (18). The actual amount of these (or other) lexical features influencing eye movement parameters in natural reading of literary texts is a wide-open empirical question. These complex units then are combined to larger units like phrases, sentences, stanzas or paragraphs which again are characterized by an overabundance of text features (14, 19) including a great variety of rhetorical devices (20). While it is far from easy to qualitatively describe all these features—as evidenced by extensive debates on e.g., the classification of metaphors and similes (21)—, the challenge to quantify relevant text features properly is even greater and still in its beginnings. To start empirical investigations using (more) natural and complex materials, appropriate models and methods are necessary to handle the plethora of text and/or reader features and their multiple (nonlinear) interactions. On the modeling side, the Neurocognitive Poetics Model of literary reading (NCPM) (22, 14, 23 ,24, 25, 26) is a first theoretical account offering predictions about the relationship between different kinds of text features and reader responses, e.g., in eye tracking studies using natural text materials (27, 28). On the methods side, inspired by the NCPM, our group has been working for quite some time on different QNA approaches. In contrast to qualitative analysis, these try to quantitatively describe a maximum of the psycholinguistic features of complex natural verbal materials, as impressively demonstrated using the example of the 154 Shakespeare sonnets (1). Additionally, this approach proposes advanced tools for computing both cognitive and affective-aesthetic features potentially influencing reader responses at all three levels of observation, i.e., the experiential (e.g., questionnaires and ratings (29, 30, 31, 32, 1, 33, 34, 35)), the behavioral (e.g., eye movements(2)), and the neuronal (36).

Shakespeare’s sonnets indeed are a particularly challenging and fascinating stimulus material for QNA and count among the most aesthetically successful or popular pieces of verbal art in the world. Facilitating QNA, most of them have the same structure and rhythmic pattern, typically decasyllabic 14-liners in iambic pentameter with three quatrains and a concluding couplet, making them perfect research materials. They have been the object of countless essays by literary critics and of theoretical scientific studies (37, 38, 39). Furthermore, all 154 sonnets have been extensively ‘QNA-ed’ in our previous work yielding precise predictions concerning e.g., eye movement data (1). Furthermore, to our knowledge, none of the previous studies on reading literary texts or poems (40, 41, 32, 28, 42, 43, 44) examined the eye movement behavior of Shakespeare sonnets.

Since it is not possible to identify all relevant features characterizing a natural text [e.g., over 50 features mentioned for single word recognition (18) or over 100 features computed for the corpus of Shakespeare sonnets (1)], nearly all empirical studies we know of tested only a few selected features while ignoring the others without giving explicit reasons for this neglect, e.g., by using eye tracking (45, 46, 47, 48, 49, 10). Thus, for the present study about the influence of basic psycholinguistic features we decided to start –relatively– simple by concentrating on a set of seven easily computable (sub)lexical surface features combining well established and less tested ones. We excluded complex inter- and supralexical features (e.g., surprisal, syntactic simplicity), as well as any features that cannot be computed via QNA (e.g., age-of-acquisition, metaphoricity). The resulting set of surface features consists of two standard features (*word length*, *word frequency*) used in many eye movement studies and three standard features from word recognition studies much less used in the eye movement field (*orthographic neighborhood density*, *higher frequent neighbors*, and *orthographic dissimilarity*), and two phonological features theoretically playing a role in poetry reading (*consonant vowel quotient*, *sonority score*). In the following paragraphs, we further explain these features and summarize their effects, if available, observed in eye tracking studies using single sentences or short nonliterary texts:

In eye tracking studies of reading non-literary texts it is widely acknowledged that longer and low frequency words attract longer total reading time (sum of all fixations on the target word) and more fixations (50, 51, 52 ,53). Apart from these two basic surface features, a wealth of research also found effects of *orthographic neighborhood density* (number of words that can be created by changing a single letter of a target word (54), e.g., bat, fat, and cab are neighbors of cat) in word recognition and reading tasks (55). While effects of *orthographic neighborhood density* are usually facilitative, the presence of *higher frequent neighbors* in the hypothetical mental lexicon inhibits processing of a target word (56, 57, 58). However, there are no clear conclusions as to the effects of both features on eye movements in reading (25). Furthermore, using the Levenshtein distance metric, we can also compute an additional *orthographic dissimilarity index* for all words, going beyond the standard operationalization based on words of the same length. As far as we know, systematic effects of the above features on eye movements in the reading of poetry have not been reported so far.

Most people will agree with the statement that poetry is an artful combination of sound and meaning (21). While the above features are basically ‘orthographic’, the effects of sublexical and lexical phonological features that have been found in a variety of silent reading studies (59, 60, 61, 62, 63, 64, 23, 3, 65, 66) and the wide use of phonetic rhetorical devices in poetic language lead us to include also two phonological features: the *consonant vowel quotient* and the *sonority score*. *Consonant vowel quotient* is a simple proxy for the pronounceability of a word—which hypothetically is related to its ease of automatic phonological recoding (67). To quantify the acoustic energy or loudness of a sound, called sonority (68), we used the *sonority score*, a simplified index based on the sonority hierarchy of English phonemes, which allows to estimate the degree of distance from the optimal syllable structure (69). It was previously applied in the study of aphasia (70) and has recently been proposed as an important feature influencing the subjective beauty of words (29). There is evidence that consonant status and sonority play a role in silent reading (71, 72), especially of poetic texts (73). Both features have not been examined in literary reading studies using eye tracking.

### Non-linear Interactive Models and Predictive Modeling

With the help of QNA, we can quantify psycholinguistic features and predict reader responses successfully (34). However, we still need to tackle the second challenge: within and between the disciplines involved in reading research there is an unspoken consent that all these psycholinguistic features influence the reading and interpretation of literary texts in a highly interactive and nonlinear way (14, 23, 34, 21). Kliegl et al. (74) already pointed out that using standard accounts like hierarchical regressions is not a solution for handling intercorrelated predictors and the nonlinear relationship between predictors and reading behavior. Consequently, we must look for appropriate tools to tackle these problems. One option is offered by recent developments e.g., in the fields of bioinformatics (75), ecology (76, 77), geology and risk analysis (78, 79), quantitative sociolinguistics (80, 81), epidemiology (82), neurocognitive poetics (29, 19, 33, 34, 1), fMRI data analysis (83) or applied reading research (84, 85) highlighting the application of machine learning tools like neural nets or bootstrap forests to predictive modeling accounts of big data sets with complex interactions and intercorrelations. Moreover, as an alternative and complement to the traditional ‘explanation approach’ of experimental psychology, machine learning principles and techniques can also help psychology become a more predictive and explorative science (86, 87). Thanks to such computational methods, tackling the challenge of analyzing human cognition, emotion or eye movement behavior in rich naturalistic settings (88) has become a viable option especially as concerns literary reading (89, 90, 26).

For present study, two non-linear interactive models, i.e., neural nets and bootstrap forests, were compared with one general linear model (standard least squares regression), to find out which approach optimally predicted relevant eye movement parameters during the reading and experiencing of poetry. The neural net model is a multi-layer perceptron which can predict one or more response variables using a flexible function of the input variables. It has the ability to implicitly detect all possible (nonlinear) interactions between predictor variables and a number of other advantages over regression models when dealing with complex stimulus-response environments (82). Bootstrap forests predict a response by averaging the predicted response values across many decision trees. Each tree is grown on a bootstrap sample of the training data (91). Both the non-linear interactive models and the general linear model were evaluated in a predictive modeling approach comparing a goodness of fit index (*R^2^*) for training and validation sets.

Taken together, in the context of our QNA-based predictive modeling approach, here we considered a minimalistic first attempt at introducing an already considerably more complex way of analyzing eye movements in reading poetic texts. We focused on potential effects of seven simple ‘surface’ features: *word length*, *word frequency*, *orthographic neighborhood density*, *higher frequency neighbors*, *orthographic dissimilarity index*, *consonant vowel quotient*, and *sonority score* on three eye movement parameters (first fixation duration, total reading time and fixation probability).

### Hypotheses

Since non-linear interactive models can deal with complex interactions and detect hidden structures in complex data sets (92), we proposed that they would outperform the general linear model and produce satisfactory model fits for both the training and validation sets.

Based on previous eye tracking studies and existent models of eye movement control (48, 93, 94, 46, 49), we assumed that *word length* and *word frequency* play a key role in accounting for variance in total reading time and fixation probability, i.e., longer and low frequency words should attract longer total reading time and higher fixation probability also in poetry reading.

On account of the facilitative effect of *orthographic neighborhood density* and the inhibitory effect of *higher frequency neighbors* in the above mentioned word recognition studies, we also expected words with many (lower frequency) orthographic neighbors to produce shorter total reading time and lower fixation probability than low *orthographic neighborhood density* words and words with *higher frequency neighbors*. Similarly, we hypothesized that higher *orthographic dissimilarity* of a word (as a proxy for its orthographic salience) would increase its total reading time and fixation probability.

As concerns the two phonological features, *consonant vowel quotient* and *sonority score*, our hypothesis was that words with a high *consonant vowel quotient* (as a proxy for hindered phonological processing) and *sonority score* (as a proxy for increased aesthetic potential) require a more exigent processing (95, 71, 96) and thus would attract longer reading time and higher fixation probability. All effects were assumed to be smaller or non-significant for first fixation durations which usually reflect fast and automatic reading behavior less influenced by lexical parameters (97, 8).

## Methods

### Participants

Fifteen native English participants (five female; *M*_age_= 31.5 years, *SD*_age_ = 14.1, age range: 18–68 years) were recruited from an announcement released at Freie Universität Berlin. All participants had normal or corrected-to-normal vision. They were naive to the purposes of the experiment and were not trained literature scholars of poetry. Participants gave their informed, written consent before commencing the experiment and received either course credit or volunteered freely. This study was conducted in line with the standards of the ethics committee of the Department of Education and Psychology at Freie Universität Berlin.

### Apparatus

Participants’ eye movements were recorded with a sampling rate of 1000 Hz, using a remote SR Research EyeLink 1000 desktop-mount eye tracker (SR Research Ltd., Mississauga, Ontario, Canada). Stimulus presentation was controlled by Eyelink Experiment Builder software (version 1.10.1630, https://www.sr-research.com/experiment-builder). Stimuli were presented on a 19-inch LCD monitor with a refresh rate of 60 Hz and a resolution of 1,024 × 768 pixels. A chin-and-head rest was used to minimize head movements. The distance from the participant’s eyes to the stimulus monitor was approximately 50 cm. We only tracked the right eye. Each tracking session was initialized by a standard 9-point calibration and validation procedure to ensure a spatial resolution error of less than 0.5° of visual angle.

### Design and Stimuli

The three Sonnets chosen from the Shakespeare Corpus of 154 sonnets were: Sonnets 27 (‘Weary with toil…’), 60 (‘Like as the waves…’) and 66 (‘Tired with all these…’). The choice was made by an interdisciplinary team of experts taking into account the considerable poetic quality and representativeness of the motifs not only within the Shakespeare Sonnet’s corpus but also within European poetry. The motifs are: love as tension between body and soul (sonnet 27), death as related to time and soul (sonnet 60) and social evils during the period Shakespeare lived (sonnet 66). All three have the same metrical and rhythmical structure as most Shakespeare sonnets (see Introduction). Inspired by our previous QNA study on Shakespeare sonnets (1), we conducted a fine-grained lexical analysis of all words used in the present three sonnets, summarized in Table 1. The Pearson Chi-square test indicated no significant differences in the distribution of four main word classes between the three sonnets (*χ^2^* = 6.31, *df* = 6, *p* = .39). We therefore collapsed the data across all sonnets to increase statistical power for predictive modeling.

**Table 1.** Number of Words per Category within Each Sonnet and within all Three Sonnets

Sonnet	*Closed-class*	*Adj./ Adv.*	*N.*	*V.*	Total
	count [ % ]	count [ % ]	count [ % ]	count [ % ]	
27	49 [44.14]	20 [18.02]	28 [25.23]	14 [12.61]	111
60	48 [44.44]	12 [11.11]	30 [27.78]	18 [16.67]	108
66	33 [36.26]	20 [21.98]	21 [23.08]	17 [18.68]	91
Total	130 [41.94]	52 [16.77]	79 [25.48]	49 [15.81]	310

*Note.* *Closed-class* refers to the category of function words; *Adj.*/ *Adv.* refers to adjective or adverb; *N.* refers to noun; *V.* refers to verb; % is the percentage of each word category within each sonnet or within all three sonnets.

### Procedure

The experiment was conducted in a dimly lit and sound-attenuated room. The data acquisition for each sonnet was split in two parts: a first initial reading of the sonnet with eye tracking and a following paper-pencil memory test accompanied by several rating questions and marking tasks.

For the initial reading participants were instructed to “read each sonnet attentively and naturally” for their own understanding. Prior to the onset of the sonnet on a given trial, participants were presented with a black dot fixation marker (0.6° of visual angle), to the left of (the left-side boundary of) the first word in line 1; the distance between the cross and first word was 4.6°. The sonnets were presented to the participants automatically, when they fixated on a fixation marker presented left to the first line. Participants read the sonnets following their own reading speed. They could go back and forth as often as they wanted within a maximum time window of two minutes. Thirteen participants stopped reading before this deadline. To achieve a certain level of ecological validity, all sonnets were presented left-aligned in the center of the monitor (distance: 8.0° from the left margin of the screen) by using a variable-width font (Arial) with a letter size of 22-point size (approximately 4.5 × 6.5 mm, 0.5 × 0.7 degrees of visual). In order to facilitate accurate eye tracking 1.5-line spacing was used.

For the second part of data acquisition, participants went to another desk to work on the paper-pencil tasks self-developed in close cooperation with literature scholars. Our questionnaire had altogether 18 close- and open-ended questions concerning memory, topic identification, attention, understanding and emotional reactions. It also included three marking tasks where participants had to indicate unknown words, key words and the most beautiful line of the poem (the rating results will be reported elsewhere by the ‘humanities’ section of our interdisciplinary team). After answering the questionnaire for the first sonnet, participants continued with reading the second sonnet in front of the eye tracker and so on. The order of the three sonnets was counterbalanced across participants. In order to make the reading of the first sonnet comparable to the reading of the latter two, participants became acquainted with the questionnaire before the initial reading of the first sonnet.

At the beginning and end of the experiment, we used an English translation of the German multidimensional mood questionnaire (MDBF) (98) to evaluate the participants’ mood state. This questionnaire assesses three bipolar dimensions of subjective feeling (depressed vs. elevated, calmness vs. restlessness, sleepiness vs. wakefulness) on a 7-point rating scale. The results showed that our participants were in a neutral mood of calmness and slight sleepiness. Simple *t*-tests comparing the mood ratings at the beginning and the end of the experiments indicated no significant mood changes (all *t* (14)s < 1). Thus, reading sonnets did not induce longer-lasting changes in the global dimensions assessed by the MDBF.

**Figure 1.**
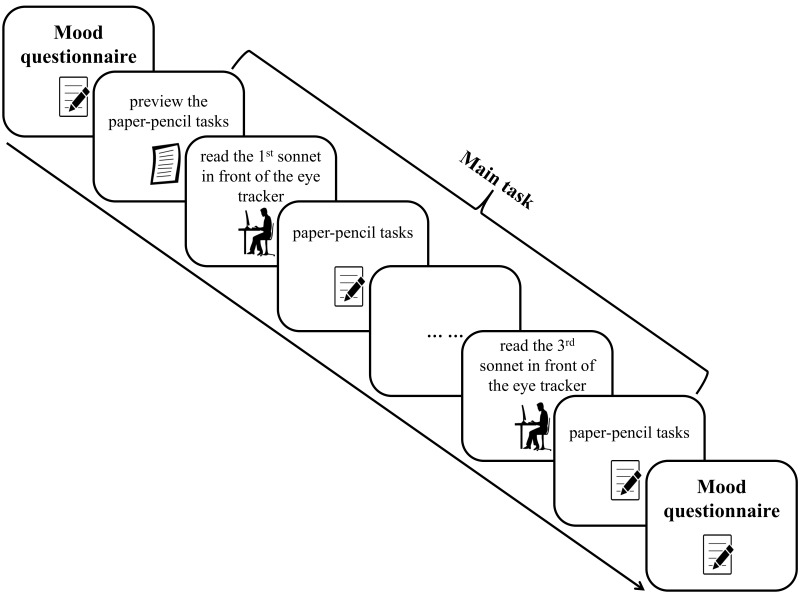
**The Procedure of the Experiment.** An English translation of the German multidimensional mood questionnaire (MDBF) (98) was presented to the participants before and after the main tasks to evaluate whether sonnets reading induced longer-lasting changes in participants’ mood state. The data acquisition for each sonnet was split in two parts: first initial reading of the sonnet with eye tracking and the following paper-pencil tasks. After answering the questionnaire for the first sonnet, participants continued with reading the second sonnet in front of the eye tracker and so on. The order of the three sonnets was counterbalanced across participants. In order to make the reading of the first sonnet comparable to the reading of the latter two, participants became acquainted with a questionnaire example before the initial reading of the first sonnet.

Altogether, the experiment took about 40 minutes (see Figure 1 for an illustration of the procedure).

### Data Analysis

**Psycholinguistic features.** All seven psycholinguistic features were computed for all unique words (word-type, 205 words, data for words appearing several times in the texts were the same) in the three sonnets based on the Gutenberg Literary English Corpus as reference (GLEC) (99): *word length* (*wl*) is the number of letters per word; *word frequency* (*logf*) is the log transformed number of occurrences of word; *orthographic neighborhood density* (*on*) is the number of words of the same length as the target word differing by one letter; *higher frequent neighbors* (*hfn*) is the number of orthographic neighbors with higher word frequency than the target word; *orthographic dissimilarity density* (*odc*) is the target word’s mean Levenshtein distance from all other words in the corpus, a metric that generalizes on to words of different lengths; *consonant vowel quotient* (*cvq*) is the quotient of consonants and vowels in one word; *sonority score* (*sonscore*) is the sum of phonemes’ sonority hierarchy with a division by the square root of *wl* (the sonority hierarchy of English phonemes yields 10 ranks: [a] > [e o] > [i u j w] > [ɾ] > [l] > [m n ŋ] > [z v] > [f θ s] > [b d ɡ] > [p t k]) (69, 34), e.g., in our three sonnets, ART got the *sonscore* of 10×1 [a] + 7×1 [r] + 1×1 [t] = 18/ SQRT (3) = 10.39.

The correlations between our seven features are given in Table 2. There were several significant correlations (e.g., *wl* & *on*, *r* = .81, *p* < .0001) indicating the usefulness of machine learning tools in literary text reading studies.

**Table 2.** Correlations between Seven QNA Features

Variables	1	2	3	4	5	6	7
1. *wl*	−						
2. *logf*	-.75	−					
3. *on*	-.81	.68	−				
4. *hfn*	-.31	.00	.36	−			
5. *odc*	.74	-.48	-.39	-.18	−		
6. *cvq*	.19	-.10	-.24	-.05	.10	−	
7. *sonscore*	.72	-.55	-.57	-.28	.62	.00	−

**Eye tracking parameters.** Raw data were pre-processed using the EyeLink Data Viewer (https://www.sr-research.com/data-viewer/)^1^. Rectangular areas of interest (AOI) were defined automatically for each word; their centers were coincident with the center of each word. For the upcoming analysis we first calculated for each word, participant and sonnet the first fixation duration (duration of first fixation on the target word) as a measure of word identification, gaze duration (the sum of all fixations on the target word during first pass), re-reading time (sum of fixations on the target word after first pass), and the total reading time (sum of all fixations on the target word) as a measure of general comprehension difficulty (100). In a next step we aggregated the data over all participants to obtain the mean values for each word within each sonnet. For this aggregation skipped words were treated as missing values (skipping rate: *M* = .13, *SD* = .04). The amount of skipping was taken into account by calculating the fixation probability for each word. Words fixated by all participants, like ‘captain’ (sonnet 66), ‘cruel’ (sonnet 60) or ‘quiet’ (sonnet 27) had a probability of 100%. Words fixated by only one or two participants like ‘to’ (sonnet 27), ‘in’ (sonnet 60), or ‘I’ (sonnet 27) had fixation probabilities below 20%. In total, over 40% of the words had a fixation probability of 100% leading to a highly asymmetric distribution. Due to the fact that our psycholinguistic features do not differ for the same word occurring at different positions within a poem all eye tracking measures were aggregated again across sonnets. For all words appearing twice or more often within all three sonnets data were collapsed into a general mean.

Before running the three different models we calculated the correlations between the five aggregated eye tracking parameters. Because gaze duration had a high correlation with first fixation duration (*r* = .56, *p* < .0001) and total reading time (*r* = .73, *p* < .0001), and regression time had a high correlation with total reading time (*r* = .97, *p* < .0001), we only chose first fixation duration, total reading time and fixation probability as response parameters in the predictive modeling analyses (see Table 3).

**Table 3:** Correlations between Five Common Eye-movement Parameters used in Reading Research

Variables	1	2	3	4	5
1. First fixation duration	−				
2. Gaze duration	.56	−			
3. Total reading time	.30	.73	−		
4. Fixation probability	.13	.31	.48	−	
5. Regression time	.16	.53	.97	.47	−

**Predictive modeling.** JMP 14 Pro (https://www.jmp.com/en_us/software/predictive-analytics-software.html) was used to run all statistical analyses^2^. The values of all variables (seven predictors and three eye movement parameters) were standardized before modeling. To counter possible overfitting, for all three models we used a cross-validation procedure using 90% of the data as training set and the remaining 10% as validation set^3^. Given the intrinsic probabilistic nature of two of the models and the limited sample size (*N* = 205 words, i.e., about 20 in the validation sets), predictive modeling results varied across repeated runs, depending on which words were selected as training or validation subset. Therefore, the procedure was repeated 1000 times and the model fit scores were averaged (77).

When the model fits of non-linear interactive tools (i.e., neural nets, bootstrap forests) were acceptable (*R^2 ^*> .30; low *SD*), feature importances (*FI*s) were calculated. *FI* is a term used in machine learning (https://scikit-learn.org/stable/modules/feature_selection.html). They were computed as the total effect of each predictor assessed by the *dependent resampled inputs* option of the JMP14 Pro software. The total effect is an index quantified by sensitivity analysis reflecting the relative contribution of a feature both alone and in combination with other features (for details(79)). This measure is interpreted as an ordinal value on a scale of 0 to 1 with *FI* values > .1 considered ‘important’ (75). To make our results better comparable with previous work, we also tested the effects of ‘important predictors’ (*FI*s > .10) in simple linear regressions using again the cross-validation procedure (90%/ 10% split) for 1000 times, although the intercorrelations between the predictors were not eliminated. If general linear model, i.e., standard least squares regression, got acceptable model fit as described above, instead of reporting *FI*s and simple regression results, we would report the mean of 1000 iterations’ parameter estimates.

We repeated the described analytical procedure for all three eye tracking parameters separately.

## Results

**Figure 2.**
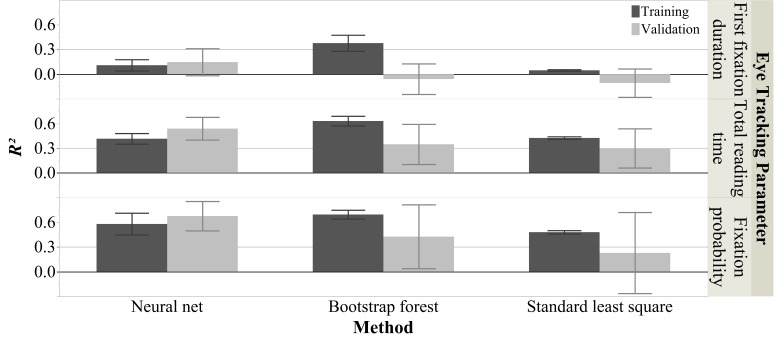
**Model Fits of Different Measure Groups via Different Modeling Methods.** This figure shows the mean *R^2^s* from 1000 iterations for three eye tracking parameters for both the training and validation sets using all three modeling approaches. Each error bar is constructed using 1 standard deviation from the mean.

**Figure 3.**
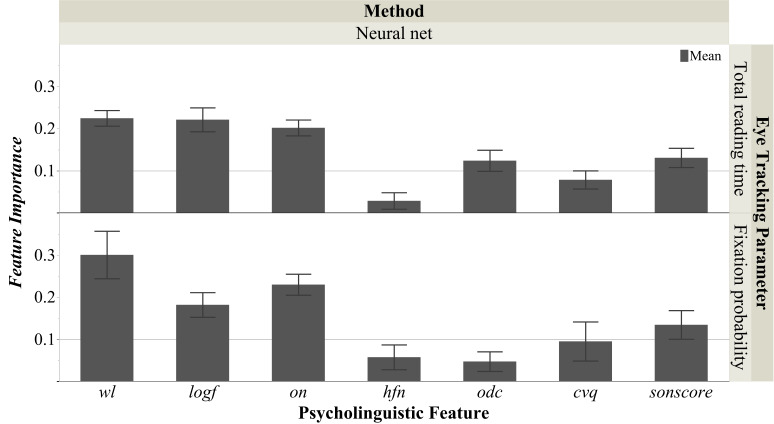
**Feature Importances for Total Reading Time and Fixation Probability.** Figure 3 shows the feature importances (*FI*s) for the neural net model. The *FI*s were calculated by using the *dependent resampled inputs* option and mean total effects of 1000 iterations. The total effect is an index quantified by sensitivity analysis, which reflects the relative contribution of that feature both alone and in combination with other features (for details (79). All seven psycholinguistic features were computed for all unique words (word-type, 205 words, data for words appearing several times in the texts were the same) in the three sonnets based on the Gutenberg Literary English Corpus as reference (GLEC) (99): *wl* was the number of letters per word; *logf* was log transformed word, *on* was the number of words of the same length as the target differing by one letter, *hfn* was the number of orthographic neighbors with higher word frequency than the target word; *odc* was the target word’s mean Levenshtein distance from all other words in the corpus; *cvq* was the quotient of consonant and vowels in one word; *sonscore* was a simplified index based on the sonority hierarchy of English phonemes which yields 10 ranks (69, 34). Each error bar is constructed using 1 standard deviation from the mean. (Note that, because of the bad model fits (see Figure 2), the *FI*s in explaining first fixation duration were excluded from this figure).

Figure 2 shows the overall mean *R^2^*s (averaged across 1000 iterations) for the three eye tracking parameters for both the training and validation sets using all three modeling approaches. Figure 3 shows the seven *FI*s for the optimal non-linear interactive approach. Below we illustrate our results for the three eye tracking parameters respectively. At the end of the results section we also reported the effects of ‘important predictors’ (*FI* > .10) in simple linear regressions.

### Mean First Fixation Duration

Figure 2 shows that while in the training set (*train*) the bootstrap forests model’s fit was satisfactory (mean *R^2^_train_* = .38, *SD* = .10), it did not generalize to the validation set (*val*) at all (mean *R^2^_val_* = -.10, *SD* = .19). The neural nets model and standard least squares regression also showed poor fits for both training (neural nets: mean *R^2^_train_* = .11, *SD* = .07; standard least squares: mean *R^2^_train_* = .05, *SD* = .01) and validation set (neural nets: mean *R^2^_val_* = .15, *SD* = .16; mean *R^2^_val_* = -.10, *SD* = .17). Thus, none of the three models seemed appropriate for predicting first fixation durations during poetry reading (at least not in the present text-reader context). Given the poor model fits, *FI*s were not calculated.

### RMean Total Reading Time

As illustrated in Figure 2, all three model fits in the training set were good (neural nets: mean *R^2^_train_* = .42, *SD* = .07; bootstrap forests: mean *R^2^_train_* = .63, *SD* = .06; standard least squares: mean *R^2^_train_* = .43, *SD* = .02). However, only the neural net model performed well for both the training and validation sets (mean *R^2^_val_* = .54, *SD* = .14), while bootstrap forests’ and standard least squares regression’s fits in the validation set were smaller and had higher standard deviations (bootstrap forests: mean *R^2^_val_* = .35, *SD* = .25; standard least squares: mean *R^2^_val_* = .30, *SD* = .24).

The *FI* analysis of the optimal neural nets model, shown in Figure 3, suggests that two of the seven features were of minor importance *(FI*s for *hfn* and *cvq* were < .10), the rest being important: *wl* (.23), *logf* (.22), and *on* (.20) turned out to be vital predictors, followed by two other less important ones: *sonscore* (.13) and *odc* (.12).

### Fixation Probability

Similar to total reading time, for fixation probability Figure 2 also shows that the fits for the training set of all three models were good (neural nets: mean *R^2^_train_* = .58, *SD* = .13; bootstrap forests: mean *R^2^_train_* = .70, *SD* = .05; standard least squares: mean *R^2^_train_* = .48, *SD* = .02). Again, only the neural nets performed well for both the training and validation sets (mean *R^2^_val_* = .68, *SD* = .18), while the model fits in the validation sets of bootstrap forests and standard least squares regression were insufficient (bootstrap forests: mean *R^2^_val_* = .43, *SD* = .39; standard least squares: mean *R^2^_val_* = .23, *SD* = .49).

For the *FI*s of neural net model shown in Figure 3, only four predictors were of importance: *wl* (.30) > *on* (.23) > *logf* (.18) > *sonscore* (.14) *(FI*s for *odc*, *hfn* and *cvq* were < .10).

### Simple linear regressions

Simple linear regression results indicate that: Words with longer *wl* (total reading time: mean *R^2^_train_* = .37, *SD* = .02; mean *R^2^_val_* = .29, *SD* = .27; fixation probability: mean *R^2^_train_* = .33, *SD* = .01; mean *R^2^_val_* = .14, *SD* = .75), lower *logf* (total reading time: mean *R^2^_train_* = .36, *SD* = .02; mean *R^2^_val_* = .25, *SD* = .26; fixation probability: mean *R^2^_train_* = .27, *SD* = .02; mean *R^2^_val_* = .06, *SD* = .66) and smaller *on* (total reading time: mean *R^2^_train_* = .26, *SD* = .01; mean *R^2^_val_* = .18, *SD* = .23; fixation probability: mean *R^2^_train_* = .33, *SD* = .02; mean *R^2^_val_* = .09, *SD* = .73) had longer total reading time and a higher fixation probability. Words with higher *odc* (total reading time: mean *R^2^_train_* = .17, *SD* = .02; mean *R^2^_val_* = .07 *SD* = .26) attracted longer total reading time. The linear relationship between *sonscore* and the two eye movement parameters was positive: total reading time: mean *R^2^_train_* = .19, *SD* = .01; mean *R^2^_val_* = .11, *SD* = .20; fixation probability: mean *R^2^_train_* = .15, *SD* = .001; mean *R^2^_val_* = .02, *SD* = .41.

## Discussion

Following up on earlier proposals (1), this study aimed to identify psycholinguistic surface features that shape eye movement behavior while reading Shakespeare sonnets by using a combination of QNA and predictive modeling techniques. Since understanding what happens while readers read poetry is a very complex task, a major challenge of Neurocognitive Poetics is to develop appropriate tools facilitating this task (23), in particular new combined computational QNA and machine learning tools (29, 33, 34). A wealth of text features can be quantified via QNA and their likely nonlinear interactive effects can best be analyzed with state-of-the-art predictive modeling techniques which can produce results largely differing from standard general linear model analyses (81, 86). Such techniques can deal with complex interactions difficult to model in a mixed-effects logistic framework (80) and detect hidden structure in complex data sets, e.g., by recursively scanning and (re-)combining variables (92).

Our results provide evidence for current theoretical discussions which highlight the good reputation regarding the predictive performance of non-linear interactive models (86, 87): both non-linear interactive models outperformed the general linear model with higher model fits (mean *R^2^*) in the training sets. Regarding the validation sets, again the general linear model performed poorly. Among the two non-linear interactive models, although bootstrap forests produced higher mean *R^2^* in the training sets, they could not generalize well to the validation set (high *SD*). The poor performance of the general linear model suggests that there are relatively large low-order (e.g., two-way) interactions or other nonlinearities that the non-linear interactive models implicitly captured but that regression did not (101, 86). The good cross-validated performance of our neural nets together with the *FI* analysis offers a considerable heuristic potential for generating hypotheses that can be tested in subsequent experimental designs. Thus, our results suggest that five out of seven surface features (*word length*, *word frequency*, *orthographic neighborhood density*, *sonority score*, and *orthographic dissimilarity index*) are important predictors of mean total reading time, while four (all previous ones minus *orthographic dissimilarity index*) are important for fixation probability, at least in the context of classical poetry.

In line with previous studies, the results from simple linear regressions indicate that longer words with lower *word frequency* and smaller *orthographic neighborhood density* attract longer total reading times and more likely fixations (50, 51, 52, 53, 55). Words with higher *orthographic dissimilarity* attract longer total reading time. Moreover, a higher *sonority* of a word increased both its total reading time and fixation probability, which is a new finding in poetry reading studies.

Our findings confirm those of previous studies in that longer and low frequency words tend to be fixated more often and longer (50, 51, 52, 53), but also suggest other important predictors, at least for the reading of poetry: words high in *orthographic neighborhood density* attract less fixations and shorter total reading time supporting the facilitative effect hypothesis of Andrews (102, 103). Additionally, words which were more *orthographically dissimilar* (i.e., more salient) also attracted longer total reading time. The results concerning the feature *higher frequent neighbors* are inconclusive across the three models which may be due to the fact that in our texts target words had relatively small *higher frequent neighbors* values (*M* = .62, *SD* = 1.24). The effect of this feature requires further investigation using different texts.

Our results also support the hypothesis that through a process of more or less unconscious phonological recoding (63, 66), text sonority may play a role in reading poetic texts: indeed, a higher *sonority* of a word increased both its total reading time and fixation probability supporting our hypothesis. Although replications—e.g. in studies with experimental designs—are required before any conclusions can be drawn, we propose that readers tend to have a more intensive phonological recoding during poetry reading (73).

In sum, we take our results as first encouraging evidence that QNA in combination with predictive modeling can be usefully applied to the study of eye tracking behavior in reading complex literary texts. We are also confident that in future studies with bigger samples (i.e., more and longer texts, more readers) and extended feature sets (including interlexical and supralexical ones (23) better generalization performance will be obtained. Here we focused on a few relatively simple QNA-based lexical surface features, but in future studies we will also use computable semantic and syntactic features at the sentence or paragraph levels, as well as predictors related to aesthetic aspects (19).

## Limitations and Outlook

A first obvious limitation of the present analyses is the focus on (sub)lexical surface features. There is little doubt that also other sublexical, lexico-semantic, as well as complex interlexical and supralexical features (e.g., syntactic complexity) affect eye tracking parameters during literary reading and, in fact, the multilevel hypothesis of the NCPM—empirically supported by behavioral, peripheral-physiological and neuronal data predicts just that (36, 32). However, for this first study with a relatively small sample size, we felt that using these seven features—several of which are novel to the field of eye tracking in reading—already made things complicated enough. We think that the present five ‘important’ features will also play a role in future extended predictive modeling studies including other features, but this is of course an open empirical question. We are currently working on extending the present research to other lexical and inter/supra-lexical features including qualitative ones like metaphoricity (104), but including more features also requires extending sample sizes (i.e., more/longer texts and more participants), a costly enterprise.

Another issue concerns the fact that word repetition or position was not included in the present analyses (i.e., data for words appearing several times in the texts were averaged). In contrast to the immediacy assumption of Just and Carpenter (50), parafoveal preview effects as predicted by current eye movement control models indicate that both spatial and temporal eye tracking parameters are affected by other factors than the features of the fixated word (for review (9, 46)). Moreover, since Just and Carpenter’s study (50), it is known that words at line beginnings or ends have a special status. This should also be true for rhyming words at line ends in sonnets or similar poem forms. While we think that our averaging procedure might have added some noise to our data without invalidating them, future studies should definitely have a closer look at word position and repetition effects in poetry reading.

Another limitation is the relatively small sample size of our study. In all, only 15 participants read only three Shakespeare sonnets with only 205 words. Even though we used predictive modeling with 1000 iterations, our findings require replication and extension. However, our goal in this study was to reach out to bridge the gap between text based qualitative analyses (dominant in the humanities) and empirical research on literature reading. In the future, we need to check the validity of our findings with larger samples and the generalizability to other literary works.

In sum, with all caution due to the limitations of this first exploratory study, the present results offer the perspective that some psycholinguistic features so far unused in (or unknown to) the ‘eye tracking in reading community’, in particular *orthographic neighborhood density* and *sonority score* could be important predictors to be looked at more closely in future research. Whether they are specific to the current selection of three sonnets or of more general interest is a valid open research issue not only for neurocognitive poetics but also for research on eye movements in reading in general.

## Ethics and Conflict of Interest

We declare that the contents of the article are in agreement with the ethics described in http://biblio.unibe.ch/portale/elibrary/BOP/jemr/ethics.html and that there is no conflict of interest regarding the publication of this paper.

## Acknowledgements

We wish to thank Giordano D., Gambino, R., Pulvirenti G., Mangen, A., Papp-Zipernovszky, O., Abramo, F., Schuster S., and Schmidtke, D. for providing and discussing some ideas in this project. We also thank Tilk S. for helping with carrying out the experiment. The authors would like to acknowledge networking support by COST Action IS1404 E-READ. Xue S. would like to thank Chinese Scholarship Council for supporting her PhD study at Freie Universität Berlin.

## Footnotes

Firstly, if fixations of a line drifted from the whole line, we corrected them into the right position. Secondly, fixation dura-tions less than 80 ms were merged with nearby fixations (if the distance between them was less than one degree) or removed from further analysis.

Based on the results of pilot and related work (e.g., (35), for the neural nets model we used the following parameter set: one hidden layer with 3 nodes, hyperbolic tan (TanH) activation function; number of boosting models = 10, learning rate = 0.1; number of tours = 10. For the bootstrap forests model, we used the default set: number of trees in the forest = 100, number of terms sampled per split = 1, minimum/maximum splits per tree = 10/ 2000, minimum size split = 5, except that we defined the max number of terms = 3. For standard least squares regression analysis, we only specified the seven fixed effects (wl, logf, on, hfn, odc, cvq, and sonscore) and predicted each eye tracking parameter using the same seven predictors (emphasis option: effect leverage).

Using a 70/30% training/test cross-validation decreased model fits, probably due to the limited sample size.
